# A method for TAT-Cre recombinase-mediated floxed allele modification in *ex vivo* tissue slices

**DOI:** 10.1242/dmm.050267

**Published:** 2023-11-03

**Authors:** Sek-Shir Cheong, Tiago C. Luis, Michelle Stewart, Rosie Hillier, Matthew Hind, Charlotte H. Dean

**Affiliations:** ^1^ National Heart and Lung Institute (NHLI), Imperial College London, London SW7 2AZ, UK; ^2^ Centre for Inflammatory Diseases, Department of Immunology and Inflammation, Imperial College London, London W12 0NN, UK; ^3^ The Mary Lyon Centre at MRC Harwell, Harwell Campus, Oxfordshire OX11 0RD, UK; ^4^ National Institute for Health Research (NIHR) Respiratory Biomedical Research Unit at the Royal Brompton and Harefield NHS Foundation Trust, London SW3 6NP, UK

**Keywords:** TAT-Cre, Cell-permeant Cre recombinase, Allele modification, Gene manipulation, Precision-cut lung slices, Precision-cut tissue slices

## Abstract

Precision-cut lung slices (PCLS) are used for a variety of applications. However, methods to manipulate genes in PCLS are currently limited. We developed a new method, TAT-Cre recombinase-mediated floxed allele modification in tissue slices (TReATS), to induce highly effective and temporally controlled gene deletion or activation in *ex vivo* PCLS. Treatment of PCLS from *Rosa26-flox-stop-flox-EYFP* mice with cell-permeant TAT-Cre recombinase induced ubiquitous EYFP protein expression, indicating successful Cre-mediated excision of the upstream *loxP*-flanked stop sequence. Quantitative real-time PCR confirmed induction of *EYFP*. We successfully replicated the TReATS method in PCLS from *Vangl2^flox/flox^* mice, leading to the deletion of *loxP*-flanked exon 4 of the *Vangl2* gene. Cre-treated *Vangl2^flox/flox^* PCLS exhibited cytoskeletal abnormalities, a known phenotype caused by VANGL2 dysfunction. We report a new method that bypasses conventional Cre-Lox breeding, allowing rapid and highly effective gene manipulation in *ex vivo* tissue models.

## Teaser

**Summary:** This paper details methodology to successfully modify floxed alleles in *ex vivo* adult tissue slices using soluble Cre, bypassing the need for Cre-Lox breeding in live animals.

## INTRODUCTION

Precision-cut tissue slices (PCTS) are an established *ex vivo* method of culturing 3D tissue slices of uniform thickness derived from human or animal organs. The use of tissue slices was first introduced in the early 20th century. However, the original model had major limitations due to the lack of slicing devices, vibrating microtomes and suboptimal incubation methods, which led to inconsistent size, thickness and viability of slices (Majorova et al., 2021). These limitations improved following the development of the Krumdieck tissue slicer (Krumdieck et al., 1980), which enables tissue to be cut at a precise, pre-defined thickness.

The PCTS technique has been applied to produce slices from different organs, including heart (Ou et al., 2020), brain (Pai and Ravindranath, 1991), kidney (de Kanter et al., 2004), liver and intestine (de Graaf et al., 2010). Using the precision cutting technique, a large number of slices can be generated from an organ, allowing the study of a wide range of conditions or variables while greatly reducing the experimental time, costs and tissue resources required. PCTS retain the overall anatomical architecture and composition of the *in vivo* organ, including appropriate cell–cell contacts, ratios and cell–extracellular matrix interactions, whereas other models, such as organoids or organ-on-a-chip, do not recapitulate the cellular and spatial complexity of the intact organ. Given the advantages of PCTS, they have become a popular 3D model that bridges the gap between *in vitro* and *in vivo* systems.

The protocols used to prepare PCTS from different organs are similar, with only slight modifications depending on the organ consistency and species used. For example, generation of precision-cut lung slices (PCLS) requires introduction of agarose into the airways to provide structural support and maintain the fragile architecture of lung tissue, during and after slicing (Placke and Fisher, 1987). In this study, we focused on lung slices, which are now routinely used for a variety of applications, including disease modelling (Alsafadi et al., 2017), drug toxicology studies (Närhi et al., 2018), host–pathogen interaction investigations (Bryson et al., 2020), injury and repair studies (Kim et al., 2021), and live imaging to visualise dynamic cellular behaviour in real time (Akram et al., 2019a). Despite the broad range of applications for which PCTS are now used, methods to manipulate genes in tissue slices remain very limited.

PCLS consist mainly of the parenchyma (gas-exchanging portion of the lungs) as well as some small airways. Within the parenchyma, there are many alveoli, each of which is composed of a layer of epithelial cells adjacent to a capillary network, enabling efficient gaseous diffusion. Although there are numerous different cell types in the adult lung parenchyma, the three key cell types that form the alveoli are endothelial cells that form the fine capillary tubes and two epithelial subtypes, alveolar type (AT)1 and AT2 cells, from which the alveolar walls are built (Guillot et al., 2013). In addition, lung fibroblasts and macrophages are integral components of the lung parenchyma. Lung fibroblasts produce extracellular matrix components to support lung structure and function, as well as play a pivotal role in orchestrating lung repair and remodelling in response to injury (White, 2015). Macrophages are key innate immune cells within the lungs that safeguard the respiratory system by helping to control the immune response against pathogens and foreign particles (Byrne et al., 2015).

Cre-Lox technology has been used extensively to generate conditional alleles and is a powerful system for introducing different types of genetic alteration – deletion, inversion, insertion and translocation – depending on the orientation of the *loxP* sites on the modified alleles (Branda and Dymecki, 2004). The Cre enzyme is used to drive the recombination of generated floxed alleles. Different methods are available to facilitate Cre expression that allow spatial or temporal control of conditional mutagenesis, including breeding with a Cre transgenic mouse, induction of modified Cre, CreERT2, by tamoxifen or 4-hydroxy-tamoxifen (4OH-T) administration (Savery et al., 2020), transfection or viral transduction of DNA plasmid-encoding Cre protein (Geoffroy and Raineteau, 2007; Rohlmann et al., 1996), and delivery of Cre mRNA (Kauffman et al., 2018). A more recently developed method employs protein-transduction domains such as TAT peptide derived from human immunodeficiency virus (HIV) TAT and a nuclear localisation sequence fused with the biologically active Cre protein to facilitate cell permeability (Peitz et al., 2002).

Cell-permeant Cre recombinase and other aforementioned Cre delivery methods have been used to successfully induce DNA recombination across different experimental settings, including cell monolayer cultures and embryo explants, including whole embryonic lungs and restricted regions of central nervous system tissue (Gitton et al., 2009; Morimoto et al., 2010; Peitz et al., 2002; Ryder et al., 2014; Savery et al., 2020). Given the versatility and uniformity of PCTS compared to organotypic explants, we hypothesised that the administration of cell-permeant TAT-Cre recombinase might be an effective method for *ex vivo* genetic manipulation in PCTS derived from floxed animals (floxed-PCTS) of any age, including adult.

Here, we demonstrate, for the first time, that TAT-Cre can effectively induce recombination of *loxP*-modified alleles without the requirement for a Cre allele, leading to successful gene activation or deletion in *ex vivo* PCLS. The TAT-Cre recombinase-mediated floxed allele modification in tissue slices (TReATS) method obviates the need for complex breeding strategies to generate animal models of interest. We anticipate that this method will be widely applicable to PCTS from other organs and will expedite the discovery of gene function, disease mechanisms and potential therapeutics.

## RESULTS

### Highly efficient TAT-Cre recombinase-mediated transgene activation in *R26R-EYFP* mouse PCLS

**Fig. 1.**
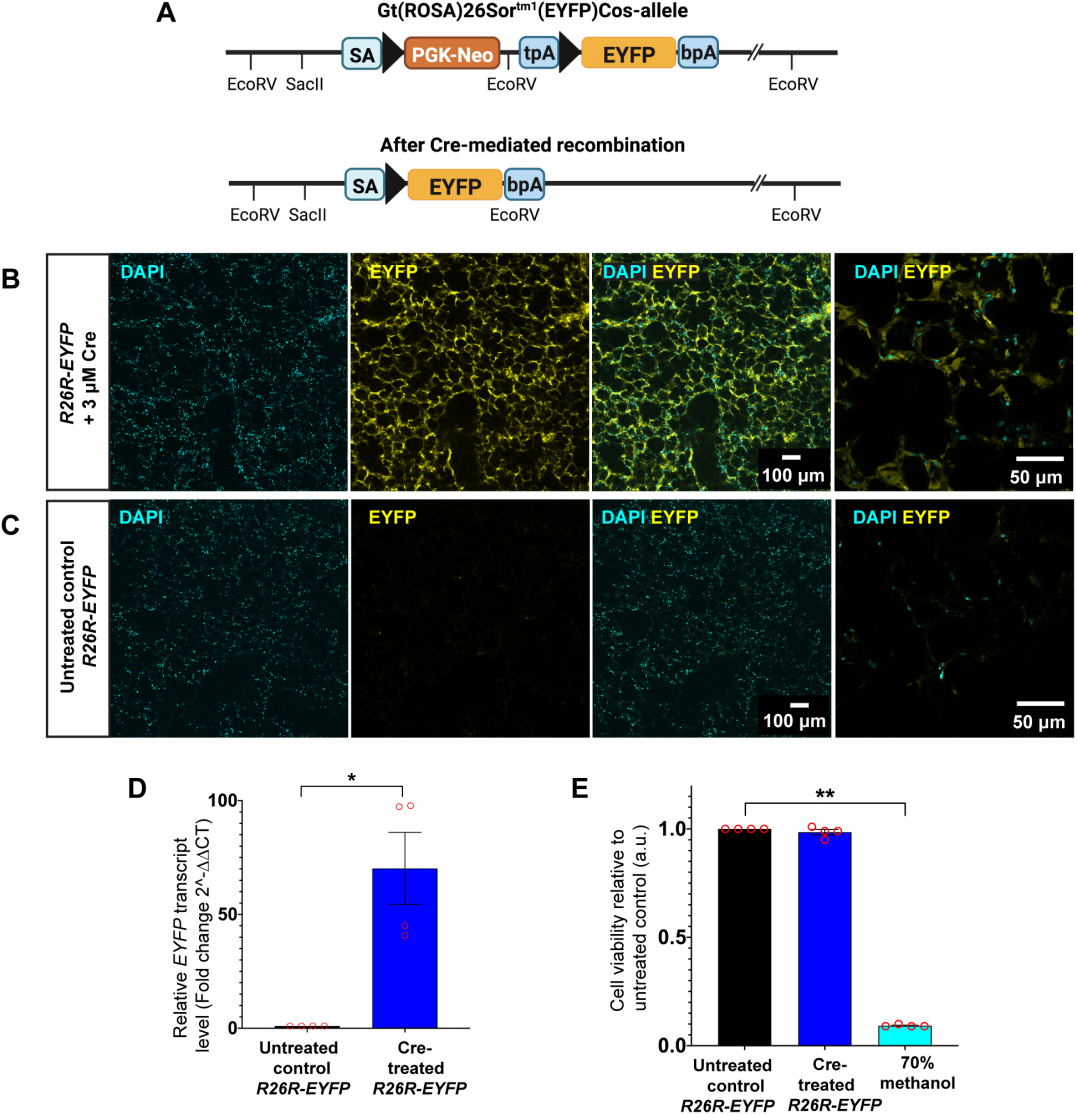
**TAT-Cre recombinase induces recombination of *loxP*-modified alleles and activates EYFP expression in precision-cut lung slices (PCLS) from adult *R26R-EYFP* mice.** (A) Schematic showing the structure of the *loxP*-modified allele in *R26R-EYFP* mice (top) and the structure of the targeted locus after Cre-mediated excision of the *loxP*-flanked (PGK-neo selective marker and a tpA transcriptional stop sequence) cassette (bottom). *EYFP* is localised downstream of the *lox-P* flanked cassette. *LoxP* sites are indicated by solid black arrowheads. (B,C) Representative images showing TAT-Cre recombinase-treated *R26R-EYFP* PCLS (B) and untreated control *R26R-EYFP* PCLS (C) analysed by fluorescence microscopy. EYFP protein expression is shown in yellow and cell nuclei were labelled with DAPI (cyan). *n*=4 mice; three PCLS per condition per experiment. Images were captured on a confocal microscope using an HC PL APO 10×/0.40 air objective lens. (D) Transcript levels for *EYFP* in untreated control and TAT-Cre recombinase-treated *R26R-EYFP* PCLS. *n*=4 mice; three PCLS were pooled for each RNA sample, with three RNA samples per condition per experiment and each experiment run in triplicate. Data are presented as mean±s.e.m.; Mann–Whitney *U-*test, *P<0.05. (E) Assessment of cell viability using MTT assay for untreated control and TAT-Cre recombinase-treated *R26R-EYFP* PCLS. PCLS treated with 70% methanol serve as a positive control for dead cells. *n*=4 mice; each experiment was run in triplicate. Data are presented as mean±s.e.m.; Kruskal–Wallis with Dunn's multiple comparisons test, **P<0.01. a.u., arbitrary units.

We first tested TAT-Cre recombinase in PCLS generated from homozygous *Rosa26-flox-stop-flox-EYFP* reporter mice (abbreviated as *R26R-EYFP* hereafter). *R26R-EYFP* reporter mice have a *loxP*-flanked stop sequence localised upstream of an enhanced yellow fluorescent protein (*EYFP*) gene inserted into the ubiquitously expressed *Gt(ROSA)26Sor* locus. In the absence of Cre recombinase activity, the transcription of *EYFP* gene is suppressed by the upstream stop codon. Upon exposure to Cre recombinase, the stop sequence is excised, allowing the expression of EYFP protein, which can readily be visualised under a fluorescence microscope (Fig. 1A).

PCLS from *R26R-EYFP* mice were incubated with 3 μM TAT-Cre recombinase in serum-free (SF) Dulbecco's modified Eagle medium (DMEM). At 72 h post-incubation, ubiquitous expression of EYFP protein was observed across PCLS (Fig. 1B), indicating successful deletion of the stop sequence upstream of the *EYFP* gene (Fig. 1A). PCLS from the same mouse cultured without TAT-Cre were used as a negative control; no EYFP was visible in these PCLS (Fig. 1C). To further validate these observations, quantitative real-time PCR (qRT-PCR) was performed on cDNA obtained from PCLS. There was a ∼70-fold increase in *EYFP* transcript levels (*P*=0.0286) in TAT-Cre recombinase-treated PCLS, compared to those in *R26R-EYFP* PCLS cultured without TAT-Cre (negative control), in which *EYFP* was undetected (Fig. 1D).

To assess whether TAT-Cre affects the viability of PCLS, 3-(4,5-dimethylthiazol-2-yl)-2,5-diphenyltetrazolium bromide (MTT) assays were conducted. TAT-Cre treatment did not result in a significant change in metabolic activity (0.99 normalised to untreated control PCLS) compared to that in untreated, control PCLS (1.00). In contrast, PCLS treated with 70% methanol, a positive control for dead cells, showed minimal metabolic activity (0.10 versus 1.00 in untreated control PCLS; *P*=0.0096) (Fig. 1E).

Furthermore, to determine whether TAT-Cre recombinase can penetrate throughout the whole tissue slice, confocal imaging was used to obtain *z*-stacks, reaching a depth of 125 μm, which corresponds to the midpoint of a 250[Supplementary-material sup1]μm-thick slice. Notably, EYFP expression was present throughout the z-stacks, indicating efficient penetrance of TAT-Cre recombinase and subsequent activation of the *EYFP* gene through the entire thickness of the PCLS (Movie 1).

### TAT-Cre recombinase-mediated *EYFP* activation in key alveolar cell types within PCLS

**Fig. 2.**
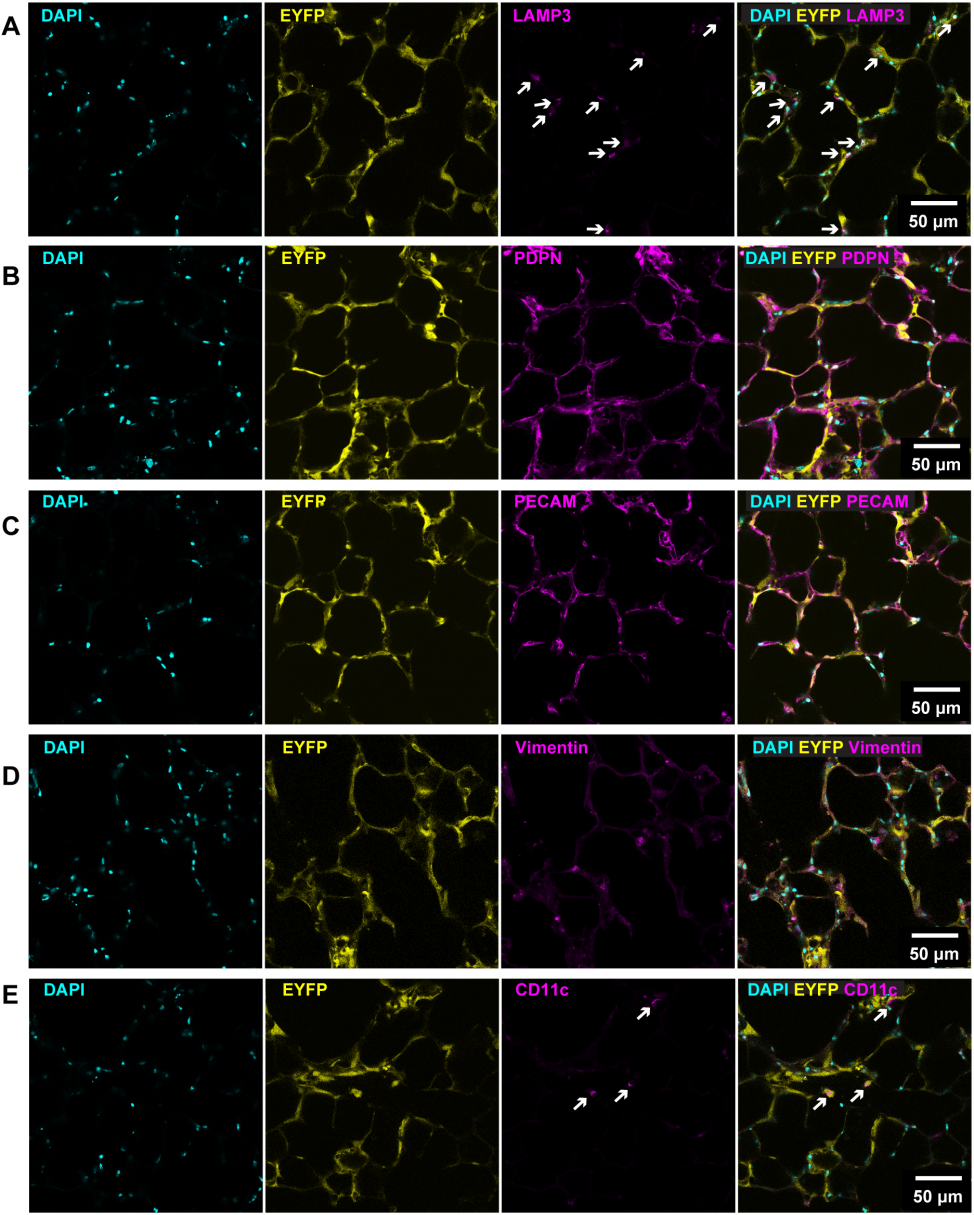
**TAT-Cre recombinase induces EYFP expression in key alveolar cell types.** (A-E) Representative images showing colocalisation of EYFP (yellow) with LAMP3^+^ differentiated cuboidal alveolar type 2 (AT2) epithelial cells (magenta; indicated by white arrows) (A), podoplanin (PDPN)^+^ alveolar type 1 (AT1) epithelial cells (magenta) (B), PECAM^+^ endothelial cells (magenta) (C), vimentin^+^ fibroblast cells (magenta) (D) and CD11c^+^ macrophages (magenta; indicated by white arrows) (E) in TAT-Cre recombinase-treated *R26R-EYFP* PCLS. Images were captured on a confocal microscope using an HC PL APO 40×/1.30 oil objective lens. Cell nuclei were labelled with DAPI (cyan). *n*=4 mice; each experiment was run in triplicate.

To identify in which alveolar cell types TAT-Cre recombinase-mediated EYFP activation occurs, TAT-Cre treated *R26R-EYFP* PCLS were immunolabelled with conjugated antibodies against previously characterised alveolar cell type-specific markers: lysosomal associated membrane protein 3 (LAMP3) for mature differentiated AT2 cells (Laresgoiti et al., 2016), podoplanin (PDPN) for AT1 cells (Marconett et al., 2017), platelet endothelial cell adhesion molecule (PECAM; also known as PECAM1) for the vascular endothelium (DeLisser et al., 2006), vimentin for fibroblast cells (Surolia et al., 2019) and CD11c (also known as ITGAX) for macrophages (Misharin et al., 2013). EYFP was present in each of these cell types (Fig. 2A-E), demonstrating that TAT-Cre recombinase treatment effectively induced *EYFP* gene activation in multiple alveolar cell populations within PCLS.

### TAT-Cre recombinase-mediated *Vangl2* deletion in *Vangl2^flox/flox^* mouse PCLS

Next, to show proof of principle that TAT-Cre could be used to drive recombination in a different floxed system, PCLS from homozygous adult *Vangl2^flox/flox^* mice were obtained to test whether TAT-Cre recombinase could be used to delete a gene of interest in tissue slices. We chose *Vangl2^flox^* mice because VANGL2 is known to regulate actin cytoskeleton remodelling (Poobalasingam et al., 2017; Yates et al., 2010; Zhang et al., 2020). We reasoned that, if successful, deletion of *Vangl2* would result in visible disruption to filamentous actin (F-actin) organisation, a phenotype that could readily be detected.

**Fig. 3.**
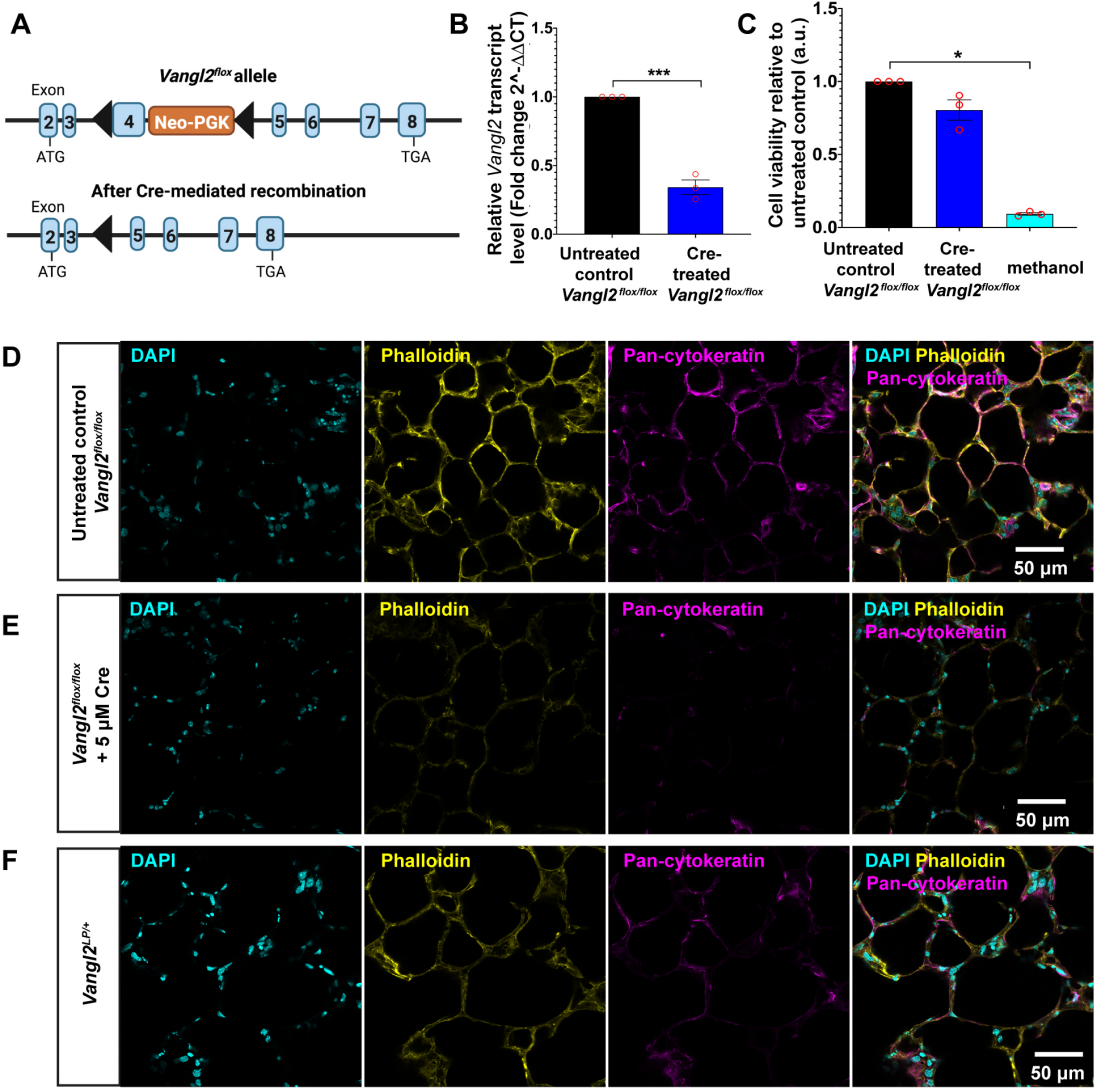
**TAT-Cre recombinase treatment induces *Vangl2* deletion in adult *Vangl2^flox/flox^* mouse PCLS.** (A) Schematic showing the structure of the *loxP*-modified allele in *Vangl2^flox/flox^* transgenic mice (top) and the structure of the targeted locus after Cre-mediated excision of the *loxP*-flanked exon 4 of the *Vangl2* gene (bottom). Deletion of exon 4 leads to loss of the critical transmembrane domains of VANGL2 protein. *LoxP* sites are indicated by solid black arrowheads. (B) Transcript levels for *Vangl2* in untreated control and TAT-Cre recombinase-treated *Vangl2^flox/flox^* PCLS using primers flanking the exon 3-4 boundary of the *Vangl2* gene. *n*=1 mouse; three PCLS were pooled for each RNA sample, with three RNA samples per condition per experiment and each experiment run in triplicate. Data are presented as mean±s.e.m.; two-tailed unpaired Student's *t*-test, ***P<0.001. (C) Assessment of cell viability using MTT assay for untreated control and TAT-Cre recombinase-treated *Vangl2^flox/flox^* PCLS. PCLS treated with 70% methanol were used as a positive control for dead cells. *n*=1 mouse; each experiment was run in triplicate. Data are presented as mean±s.e.m.; Kruskal–Wallis with Dunn's multiple comparisons test, *P<0.05. (D-F) Labelling of F-actin (Phalloidin; yellow) and immunofluorescence staining for intermediate filaments (pan-cytokeratin; magenta) in untreated control *Vangl2^flox/flox^* PCLS (D), TAT-Cre recombinase-treated *Vangl2^flox/flox^* PCLS (E) and heterozygous *Vangl2^Lp/+^* PCLS (F). Images were captured on a confocal microscope using an HC PL APO 40×/1.30 oil objective lens. Nuclei were stained with DAPI (cyan).

The *Vangl2^flox^* allele is illustrated in Fig. 3A; *loxP* sites flank exon 4 of the *Vangl2* gene. Upon exposure to Cre, recombination between the *loxP* sites results in excision of exon 4, leading to the introduction of a premature stop codon that gives rise to a truncated protein lacking the crucial trans-membrane domains and a C-terminal PDZ-binding domain that is required for interaction with other binding partners (Ramsbottom et al., 2014) (Fig. 3A). To test whether TAT-Cre could mediate recombination of the *Vangl2^flox^* allele in *ex vivo* culture, PCLS generated from a homozygous adult *Vangl2^flox/flox^* mouse were incubated with 5 μM TAT-Cre recombinase. After 72 h in culture, total RNA was extracted, and qRT-PCR was performed using primers spanning the exon 3-4 boundary of the *Vangl2* gene. Results from qRT-PCR demonstrated a significant 70% reduction (*P*<0.001) in transcript levels of *Vangl2* in TAT-Cre recombinase-treated PCLS compared with those in untreated, control PCLS from the same mouse, indicating successful TAT-Cre recombinase-mediated excision of floxed exon 4 (Fig. 3B). MTT assays demonstrated that metabolic activity in PCLS treated with 5 μM TAT-Cre recombinase was not significantly different from that in untreated, control PCLS (0.80 in Cre-treated PCLS versus 1.00 in control PCLS), indicating that cell viability was sufficiently maintained (Fig. 3C). Methanol-treated PCLS showed a significant reduction in metabolic activity, as expected (0.09 versus 1.00 in untreated, control PCLS; *P*<0.05) (Fig. 3C).

### Phenotypic characterisation of TAT-Cre-treated *Vangl2^flox/flox^* PCLS

VANGL2 is a core component of the planar cell polarity (PCP) pathway. This pathway plays a key role in driving tissue morphogenesis and repair by regulating actin cytoskeleton organisation (Poobalasingam et al., 2017; Yates et al., 2010; Zhang et al., 2020). Loss of functional VANGL2 protein is known to disrupt embryonic lung branching, alveologenesis and adult lung repair following injury, owing to dysfunctional actomyosin-driven cell migration (Cheong et al., 2020; Poobalasingam et al., 2017; Yates et al., 2010). Thus, one of the prominent phenotypes that results from VANGL2 dysfunction is highly disrupted F-actin organisation, as previously shown in a number of studies using the *Vangl2* loss-of-function (LOF) allele, *Looptail* (*Vangl2^Lp^*) (Cheong et al., 2020; Poobalasingam et al., 2017; Yates et al., 2010).

To investigate whether TAT-Cre recombinase-mediated *Vangl2* deletion in *Vangl2^flox/flox^* PCLS led to similar aberrant F-actin organisation defects, PCLS were cultured for 72 h with or without TAT-Cre and then labelled with Rhodamine Phalloidin. As expected, untreated, control PCLS from *Vangl2^flox/flox^* mice exhibited normal F-actin distribution (Fig. 3D; yellow). In contrast, F-actin was severely disrupted in *Vangl2^flox/flox^* PCLS treated with 5 μM TAT-Cre recombinase (Fig. 3E; yellow). Notably, this phenotype was more profound than that in PCLS from adult heterozygous *Vangl2^Lp/+^* mutants (Fig. 3F; yellow). Homozygous *Vangl2^Lp/Lp^* mice are not viable beyond the perinatal stage, precluding any comparison between Cre-treated *Vangl2^flox/flox^* PCLS and adult homozygous *Vangl2^Lp/Lp^* PCLS.

VANGL2 dysfunction has previously been shown to hamper traction force generation and mechanosignalling owing to defects in actomyosin contractility (Cheong et al., 2020). Perturbation of cytoplasmic intermediate filaments (IFs) is also known to impact mechanical integrity, causing reduced cell stiffness (Sanghvi-Shah and Weber, 2017). This prompted us to speculate that *Vangl2* deletion can affect IFs. To investigate the effect of *Vangl2* deletion on IFs, Cre-treated and untreated, control *Vangl2^flox/flox^* PCLS were immunostained with pan-cytokeratin, a marker for epithelial cell IFs. Interestingly, Cre-treated *Vangl2^flox/flox^* PCLS displayed highly aberrant IFs (Fig. 3E; magenta) compared with those from untreated, control PCLS from the same mouse (Fig. 3D; magenta), indicating that loss of VANGL2 perturbed IF distribution. Notably, similar, albeit less severe, abnormalities in IFs were also observed in heterozygous *Vangl2^Lp/+^* PCLS (Fig. 3F; magenta).

**Fig. 4.**
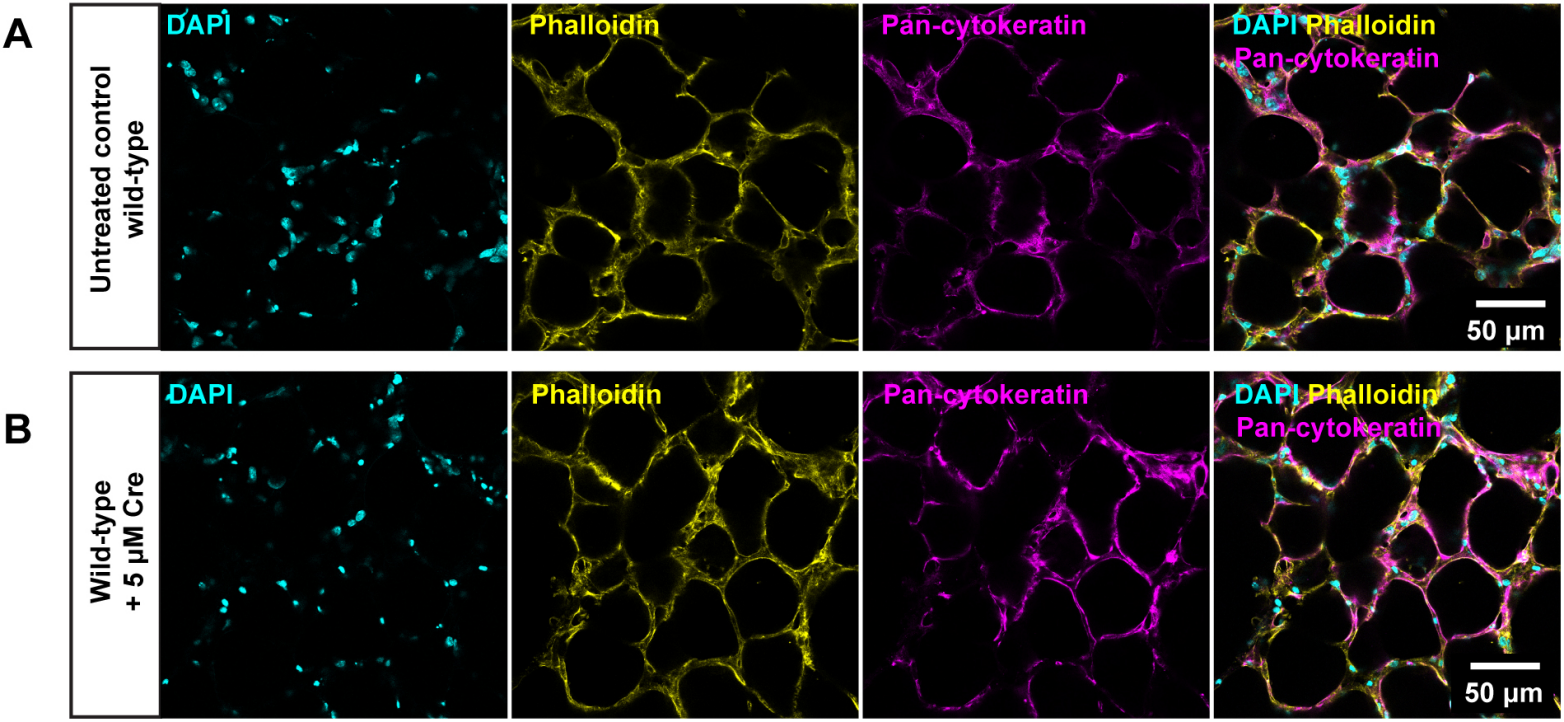
**F-actin and intermediate filament phenotype in wild-type control PCLS.** (A,B) Labelling of F-actin (Phalloidin; yellow) and immunofluorescence staining for intermediate filaments (pan-cytokeratin; magenta) in untreated control wild-type PCLS (A) and TAT-Cre recombinase-treated wild-type PCLS (B). Images were captured on a confocal microscope using an HC PL APO 40×/1.30 oil objective lens. Nuclei were stained with DAPI (cyan). *n*=3 mice; each experiment was run in triplicate.

To confirm that the observed phenotypes in F-actin and IFs were a consequence of *Vangl2* disruption rather than arising from adverse effects of TAT-Cre recombinase, the same dosage of TAT-Cre recombinase was used to treat PCLS from wild-type mice. Fig. 4A shows the presence of normal F-actin (yellow) and IFs (magenta) in untreated PCLS from wild-type mice, which were indistinguishable from those in wild-type PCLS treated with 5 μM TAT-Cre recombinase (Fig. 4B). This finding indicates that F-actin and IF anomalies observed in TAT-Cre recombinase-treated *Vangl2^flox/flox^* PCLS were due to the loss of functional VANGL2 protein and were not a result of toxic or off-target effects from exogenous Cre treatment.

## DISCUSSION

PCLS are an invaluable *ex vivo* model that closely recapitulates the complexity of the native lung environment (Alsafadi et al., 2020). Although *in vivo* models offer greater biological relevance, data generated from *in vivo* studies are often complex to interpret owing to the presence of multiple variables. Moreover, the number of animals required for *in vivo* experiments is considerable, with associated ethical and financial implications. PCLS, on the contrary, serve as an excellent intermediate model, bridging the gap between *in vitro* and *in vivo* models. However, until now, a major limitation of tissue slice models has been the lack of effective methods for genetic manipulation within *ex vivo* tissue. Currently, the only established genetic modification method for PCLS involves gene silencing through passive transfection of siRNA, which comes with drawbacks such as transient gene silencing effects and low transfection efficiency (Ruigrok et al., 2017). Thus, the development of a technique allowing permanent and irreversible gene deletion or activation within PCLS greatly enhances the versatility of this model, broadening the spectrum of studies that will be feasible with this platform.

**Fig. 5.**
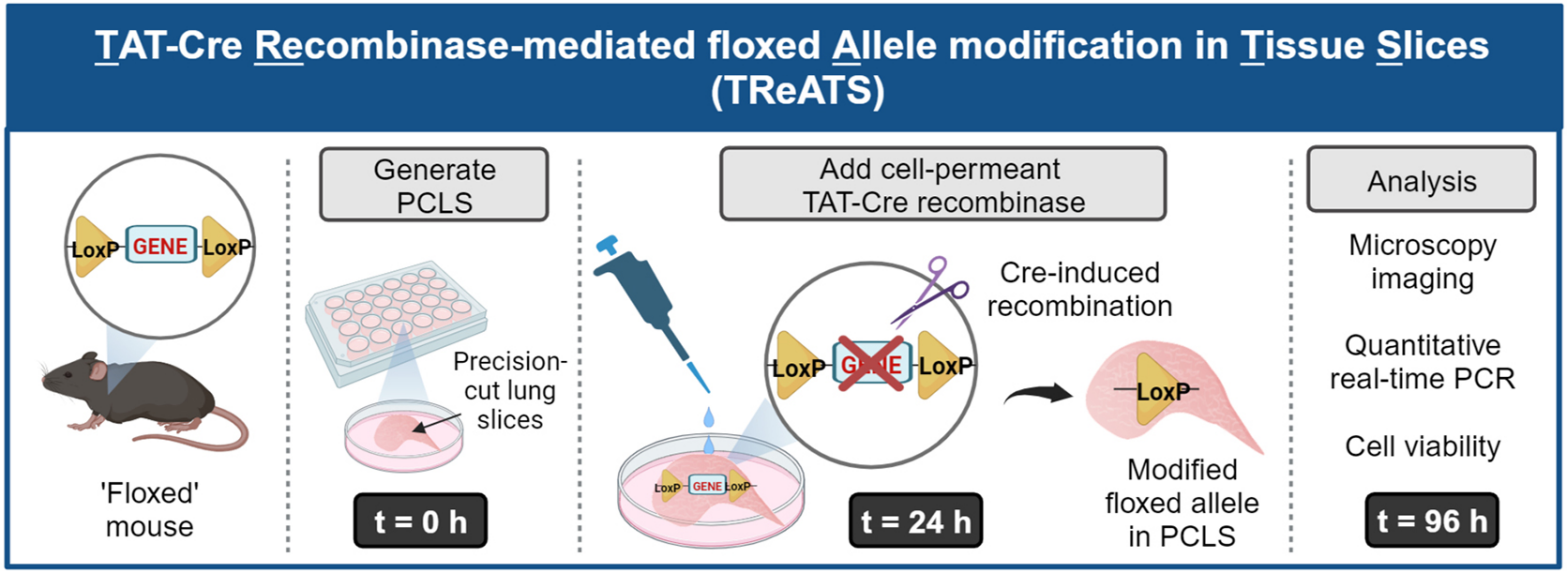
**The experimental flow of TAT-Cre recombinase-mediated floxed allele modification in tissue slices (TReATS).** PCLS are generated from a transgenic mouse carrying the floxed allele(s) of interest and maintained in DMEM. At *t*=24 h, TAT-Cre recombinase is added and PCLS are incubated in TAT-Cre solution for 72 h. At the end point (*t*=96 h), microscopy imaging and quantitative real-time PCR are performed to assess recombination efficiency. A cell viability test is conducted to determine the cell metabolic activity of PCLS. Diagram was created with BioRender.

Previously, several methods have been employed to induce Cre-mediated recombination of *﻿loxP*-modified alleles. However, each of these methods has limitations, such as leakiness of the Cre system *in vitro* or *in vivo* (Fuhrmann-Benzakein et al., 2000), costly and laborious breeding strategies, risk of toxicity caused by Cre-inducing agents such as 4OH-T (Denk et al., 2015; Savery et al., 2020), low transfection efficiencies of Cre plasmids or mRNA resulting in low recombination, instability of mRNA (Bugeon et al., 2017; Van Den Plas et al., 2003), and insertional mutagenesis caused by viral transduction (Peitz et al., 2002). To overcome these issues, this study demonstrates a new approach using cell-permeant TAT-Cre recombinase for efficient *loxP*-modified allele modification in adult *ex vivo* 3D tissue slices, TReATS (Fig. 5).

This method offers several advantages. First, Cre protein administration in PCLS bypasses complex crossbreeding strategies involving Cre transgenic mice, considerably reducing the number of animals required for experiments, in line with the 3Rs principles (Replacement, Refinement and Reduction of animals in research) (Hubrecht and Carter, 2019), as well as greatly reducing experimental time and cost.

Second, it provides a rapid solution to overcome the issue of embryonic or perinatal lethality. Mice carrying LOF allele(s) of genes required for development often die at or around birth. For example, homozygous LOF mutants of *Vangl2* (and other PCP pathway genes such as *Wnt5a* and *Ror2*) die *in utero* or perinatally owing to severe neural tube defects (Oishi et al., 2003; Yates et al., 2010; Yin et al., 2012), limiting studies in adulthood to heterozygous mutants only. In cases in which a gene plays a critical role in the function of a vital organ or system, even conditional gene deletion within specific cell populations might not prevent lethality. Conditional deletion of *Vangl2, Wnt5a* or *Ror2* in targeted lung cell populations, *Sox9^Cre/+^* lung distal epithelium, *Dermo^Cre/+^* or *Pdgfra^Cre/+^* lung mesenchyme all resulted in postnatal or perinatal lethality (Zhang et al., 2020). Although inducible systems such as doxycycline (tTA and rtTA) and tamoxifen-inducible CreERT/loxP offer an alternative way to enable gene manipulation in a temporally or spatially controlled manner to prevent lethality due to early or global ablation or overexpression, they involve complex animal breeding strategies (Rawlins and Perl, 2012). Thus, direct administration of TAT-Cre protein in tissue slices provides a simple, rapid, cost- and time-effective way of achieving temporally controlled gene manipulation directly in *ex vivo* tissue.

Another advantage is that numerous PCLS (∼100 PCLS) can be generated from the lungs of a single adult transgenic floxed animal, enabling untreated PCLS from the same animal to be incorporated into experiments that serve as a ‘true’ experimental controls, compared to the various littermate controls of different genotypes that are required for conventional Cre-Lox experiments. The ability to use controls from the same animal eliminates the potential introduction of confounding variables associated with using separate experimental and control animals. The TReATS method can also be used to vary the level of gene manipulation by applying TAT-Cre to PCLS from floxed heterozygous animals to induce single allelic gene alteration or floxed homozygotes for bi-allelic gene modification.

Furthermore, although spatial or temporal control of Cre expression using cell type-, tissue- or developmental stage-specific promoters is possible, the prerequisite and limitation of this approach is the availability and specificity of relevant promoters (Kim et al., 2018; Rawlins and Perl, 2012). Tremendous progress has been made to establish different cell- or tissue-specific promoters to restrict Cre expression in cells or tissues of interest (Kim et al., 2018; Rawlins and Perl, 2012). In addition, Cre driver lines that enable global deletion of floxed genes are available (Ramsbottom et al., 2014; Schwenk et al., 1995). However, there are currently no organ-specific promoters, i.e. Cre driver lines that can simultaneously induce recombination of floxed alleles in all cell types or tissues within a single organ, whereas the TReATS method can achieve this.

Nonetheless, the TReATS method described here is not without its limitations. One limitation is the viability of PCLS over time, in culture. In this study, we maintained the PCLS up to 96 h without significant reduction in cell viability. Other studies have shown viable PCLS for up to 8 days in culture (Alsafadi et al., 2020; Michalaki et al., 2022). If prolonged culture of PCLS is required to observe long-term effects of gene ablation or overexpression, embedding PCLS in hydrogel has been shown to effectively extend their viability for at least 3 weeks (Bailey et al., 2020).

In the current study, we validated the loss of functional VANGL2 protein following TAT-Cre recombinase treatment of *Vangl2^flox/flox^* PCLS by showing characteristic disrupted F-actin distribution in Cre-treated *Vangl2^flox/flox^* PCLS. Disorganisation of the actin cytoskeleton is a hallmark of *Vangl2* dysfunction that has previously been demonstrated in *Vangl2* LOF mouse models and following siRNA knockdown of *VANGL2* in human alveolar adenocarcinoma cells (Cheong et al., 2020; Poobalasingam et al., 2017; Yates et al., 2010). Cre-mediated recombination was assessed by qRT-PCR of the gene of interest (Fig. 1D and Fig. 3B) and visualisation of EYFP protein expression by fluorescence microscopy (Fig. 1B and Fig. 2A-E). An alternative option to assess the efficiency of *Vangl2* allele recombination would have been to evaluate VANGL2 protein levels by immunostaining or western blotting; however, it is very challenging to obtain VANGL2 protein data owing to a lack of effective and/or specific antibodies (Belotti et al., 2012), but this is a viable alternative to evaluate recombination efficiency of other targets of interest. In the future, flow cytometry could be explored as an alternative to immunostaining for validation and quantification of Cre-mediated recombination efficiencies in tissue slices (Michalaki et al., 2022).

The new strategy, TReATS, described in this paper combines two powerful technologies: PCTS and cell-permeant Cre. TReATS provides a rapid and highly effective gene manipulation solution in *ex vivo* tissue slices, which has not been possible thus far. The establishment of this method constitutes a major advance in the use of PCTS and is anticipated to greatly expand their utility for research and drug screening purposes.

## MATERIALS AND METHODS

### Mice

﻿All animal maintenance and procedures were conducted in compliance with the requirements of the Animal (Scientific ﻿Procedures) Act 1986. Animal work was approved by the South Kensington Animal Welfare and Ethical Review Body committee at Imperial College London. Mice were housed in specific pathogen-free conditions and given food and water *ad libitum*. Wild-type mice were purchased from Charles River Laboratories (UK); *R26R-EYFP* mice, previously described (Srinivas et al., 2001), were kindly gifted by Dr Luis Tiago (Imperial College London, UK); and *Vangl2^flox/flox^* and *Vangl2^Lp/+^* mice were supplied by Medical Research Council (MRC) Harwell (Oxford, UK). *R26R-EYFP*, *Vangl2^flox/flox^ and Vangl2^Lp/+^* mouse strains were genotyped using previously described methods (Ramsbottom et al., 2014; Srinivas et al., 2001; Strong and Hollander, 1949). The structures of floxed alleles in *R26R-EYFP* and *Vangl2^flox/flox^* are illustrated in Fig. 1A and Fig. 3A, respectively. *Vangl2^Lp/+^* mice carry a heterozygous missense mutation S464N that results in VANGL2 LOF (Kibar et al., 2001; Murdoch, 2001). Wild-type, *R26R-EYFP* and *Vangl2^flox/flox^* were maintained on a C57BL/6J background, whereas *Vangl2^Lp/+^* mice were of C3H/HeH strain. Male or female adult mice aged 10-20 weeks were used. *R26R-EYFP* (*n*=4), *Vangl2^Lp/+^* (*n*=4), wild-type (*n*=3) and *Vangl2^flox/flox^* (*n*=1) mice were used in this study. All experiments were run in triplicate.

### PCLS generation and culture

﻿PCLS were generated from wild-type, *R26R-EYFP*, *Vangl2^flox/flox^* and *Vangl2^Lp/+^* adult mouse lungs as previously described with slight modifications (Akram et al., 2019b). Briefly, adult mice were humanely culled using intraperitoneal injection with pentobarbital. The anterior chest and neck wall were removed. A small incision was made in the anterior wall of the trachea just below the cricoid cartilage. A 21G rigid metal cannula was inserted into the incision, and the lungs were inflated by injecting 1.2-1.5 ml of 2% (w/v) low-gelling temperature agarose (Sigma-Aldrich, A9414) dissolved in 1× Hank's balanced salt solution (HBSS; Life Technology, 14025-100) containing 1% 4-(2-hydroxyethyl)-1-piper-azineethanesulfonic acid (HEPES) buffer (Life Technology, 15630080). After inflation, agarose was solidified by applying ice to the chest cavity. The lungs and heart were then excised from the body and immediately submerged in ice-cold HBSS/HEPES buffer and kept on ice until slicing.

Individual lung lobes were carefully separated and cut transversely at 250 µm using an automated vibratome (Precisionary Instruments, Compresstome VF-300-0Z). Slices were generated in ice-cold HBSS/HEPES buffer and transferred to 48-well plates containing ice-cold SF-DMEM (Sigma-Aldrich, 31966021) with 1% penicillin-streptomycin (Merck, P0781). PCLS were generated from the central two-thirds of the lobe to ensure consistency in size. PCLS were then incubated overnight at 37°C in 5% CO_2_ and washed three times with warm SF-DMEM to remove excess agarose before use in experiments.

### Cell-permeant TAT-Cre recombinase treatment

PCLS generated from *R26R-EYFP* mouse lungs were incubated with 3 µM TAT-Cre recombinase (Merck, SCR508) in SF-DMEM for 24 h at 37°C in 5% CO_2_. After 24 h of incubation, TAT-Cre recombinase solution was removed and fresh SF-DMEM medium was added, incubating at 37°C in 5% CO_2_ for a further 48 h. For *Vangl2^flox/flox^*, 5 µM TAT-Cre recombinase was added and PCLS were left submerged in the TAT-Cre solution for 72 h. Untreated, control PCLS from the same mouse were used as a negative control for each experiment. PCLS from wild-type mice with matched C57BL/6J background were treated with 5 µM TAT-Cre recombinase and used as a negative control.

### Live staining of PCLS and confocal imaging

At 72 h post-Cre treatment, PCLS from *R26R-EYFP* mice were immunolabelled with markers specific to key alveolar cell types using conjugated antibodies. Briefly, PCLS were incubated for 2 h at 37°C in the dark with Alexa Fluor^®^ 647-conjugated rat anti-LAMP3 (Dendritics, DDX0192A647; 1:250), podoplanin eFluor^®^ 660 (Life Technologies, 50-5381-82; 1:500), Alexa Fluor^®^ 647-conjugated PECAM (BioLegend, 102416; 1:200), Alexa Fluor^®^ 647-conjugated CD11c (BioLegend, 117312; 1:200) or BioTracker™ TiY Vimentin Live Cell Dye (Sigma-Aldrich, 1:2000) diluted in SF-DMEM (antibody and cell dye details are provided in Table S1). Cell nuclei were labelled with 4′,6-diamidino-2-phenylindole (DAPI; Life Technologies, 62248; 1:500) during the last 15 min of antibody incubation. ﻿To validate the absence of any non-specific binding of the conjugated fluorophores employed in live-cell imaging, PCLS were stained with appropriate isotype IgG control antibodies: ﻿Armenian hamster IgG-PE (BioLegend, 400907) for TiY Vimentin or rat IgG-Alexa Fluor 647 (BioLegend, 400526) for LAMP3, PDPN, PECAM and CD11c, at 1:200 for 2 h at 37°C and counterstained with DAPI as above. PCLS were then washed three times with warm Phenol Red-free SF-DMEM (Life Technologies, 21063029) and immediately used for live imaging. PCLS from *R26R-EYFP* mice were placed into uncoated 24-well µ-plates (Ibidi, 82421) and held in place by a 6.5 mm Transwell^®^ with 0.4 µm pores (Scientific Laboratory Supplies, 3470) as previously described (Akram et al., 2019b). PCLS were imaged on a Leica SP8 inverted confocal microscope using an HC PL APO 10×/0.40 air objective lens or HC PL APO 40×/1.30 oil objective lens.

To generate *z*-stack images, TAT-Cre recombinase-treated *R26R-EYFP* PCLS were transferred to an eight-well chamber microscopy slide (Ibidi, 80826) and labelled with DAPI for 15 min at 37°C. PCLS were then washed with Phenol Red-free SF-DMEM and incubated with ProLong™ Live Antifade Reagent (Invitrogen, P36975; 1:100 dilution in Phenol Red-free SF-DMEM) for 15 min to prevent fluorophore bleaching during the imaging process. Excessive ProLong™ Live Antifade solution was removed; only droplets of Antifade solution were retained in the chamber slide to immobilise PCLS and ensure that the PCLS were kept moist throughout the imaging process. A total of 126 images were captured along the *z*-plane of the PCLS (*z*=125 µm; 1 µm step size) on a Leica SP8 inverted confocal microscope using an HC PL APO 40×/1.30 oil objective lens. For some images, channel colours were changed during image post-processing for optimal data visualisation.

### Immunofluorescence and confocal imaging

PCLS from *Vangl2^flox/flox^*, *Vangl2^Lp/+^* and wild-type mice were fixed with 4% (v/v) paraformaldehyde (PFA) for 15 min at room temperature (RT), washed three times in PBS, permeabilised with 0.5% Triton X-100 in PBS at RT for 30 min, followed by 1 h blocking with PBSBT (1% bovine serum albumin, 0.2% Triton X-100 in PBS) at RT. After blocking, PCLS were incubated with mouse anti-pan-cytokeratin (Sigma-Aldrich, C2931; 1:200) diluted in PBSBT blocking buffer at 4°C overnight. After three washes in PBSBT, PCLS were then incubated with Rhodamine Phalloidin (Biotium, 00027; 1:200) and goat anti-mouse IgG (H+L) Alexa Fluor 647 secondary antibody (Thermo Fisher Scientific, 21235; 1:500) at RT for 2 h (antibody details are provided in Table S1). After washing in PBS, cell nuclei were labelled with DAPI (Sigma Aldrich, D9542; 1:500). Coverslips were then mounted with ProLong™ Gold Antifade Mountant (Invitrogen, P36930). PCLS were imaged on a Leica SP8 inverted confocal microscope using an HC PL APO 40×/1.30 oil objective lens. For some images, channel colours were changed during image post-processing for optimal data visualisation.

### RNA extraction and qRT-PCR

PCLS homogenisation was carried out using a FastPrep-24™ Tissue Homogeniser (MP Biomedicals) followed by total RNA extraction from PCLS using an RNeasy mini kit (Qiagen) according to the manufacturers’ protocols. Each RNA sample was produced by pooling three PCLS, and three RNA samples were prepared for each condition per experiment. RNA concentration and quality were assessed using a TapeStation 2200 (Agilent). Only samples with RNA integrity number (RIN) value >8 were used for cDNA conversion and subsequent qRT-PCR. Approximately 200 ng total RNA was reverse transcribed to cDNA using a High-Capacity cDNA Reverse Transcription kit (Applied Biosystems) according to the manufacturer's instructions. qRT-PCR was performed using TaqMan Fast Advanced Master Mix (Life Technologies) and run on a 7500 Fast Real-Time PCR system (Applied Biosystems). *B2m* was used as a reference gene. Relative transcript levels were analysed using the 2^−ΔΔCT^ method. Four *R26R-EYFP* and one *Vangl2^flox/flox^* mice were used, and all samples were tested in triplicate. All primers used in this study were purchased from Life Technologies as follows: *B2m* (Mm00437762_m1), *Vangl2* (Mm00473768_m1) and *YFP* (Mr04097229_mr).

### MTT assay

MTT assay (Roche, 11465007001) was performed according to the manufacturer's instructions to assess cell viability at the end point of TAT-Cre recombinase experiments. Briefly, PCLS of equal size were placed into 48-well plates, one slice per well. Then, 250 µl of 10% MTT solution in SF-DMEM was added to each well and incubated at 37°C for 45 min. MTT solution was discarded, 250 µl dimethyl sulfoxide was added to solubilise the formazan crystals that formed within the viable cells, and the PCLS were incubated for 10 min at 37°C. Then, 200 µl eluted formazan solution from each well was transferred to a 96-well plate, and absorbance (optical density) was measured at 570 nm and 690 nm using a SpectraMax^®^ iD3 microplate reader (Molecular Devices). PCLS treated with 70% methanol were used as positive control for dead cells with no metabolic activity, whereas untreated, control PCLS were used as positive control with normal metabolic activity. MTT assay was performed in triplicate for each condition.

### Statistical analysis

All graphs were produced and statistical tests were performed in GraphPad Prism 8. Data are presented as mean±s.e.m. Comparisons of multiple groups were performed using a Kruskal–Wallis test with Dunn's multiple comparison post-test. Datasets comparing two groups were analysed using a Mann–Whitney *U*-test. *P*<0.05 was considered statistically significant. Details of the statistical tests used, *n* values and number of experiments performed are provided in the figure legends.

## Supplementary Material

10.1242/dmm.050267_sup1 Supplementary information
