# Supplementary material for: A method for TAT-Cre recombinase-mediated floxed allele modification in ex vivo tissue slices
Source: Dis Model Mech. 2023 Nov 3;16(11):dmm050267. doi: 10.1242/dmm.050267 (PMC10629676; doi:10.1242/dmm.050267)
Supplement: Supplementary information [file dmm-16-050267-s1.pdf]

Table S1. List of antibodies and cell dyes used in this study.

**Primary Antibodies**	**Host**	**Conjugation**	**Dilution**	**Supplier**	**Reference**
LAMP3	Rat	Alexa Fluor® 647	1:250	Dendritics	DDX0192A647
Podoplanin	Syrian hamster	eFluor™ 660	1:500	Life Technologies	50-5381-82
PECAM	Rat	Alexa Fluor® 647	1:200	Biolegend	102416
CD11c	Armenian hamster	Alexa Fluor® 647	1:200	Biolegend	117312
Pan-Cytokeratin	Mouse	Unconjugated	1:200	Sigma-Aldrich	C2931
**Cell dyes**	**Host**	**Conjugation**	**Dilution**	**Supplier**	**Reference**
Vimentin	NA	TiY	1:2,000	Sigma-Aldrich	SCT059
Phalloidin	NA	Rhodamine	1:200	Biotium	00027
**Secondary Antibodies**	**Host**	**Conjugation**	**Dilution**	**Supplier**	**Reference**
α-mouse	Goat	Alexa Fluor® 647	1:500	Thermo Fisher Scientific	21235
IgG isotype control	Armenian hamster	PE	1:200	Biolegend	400907
IgG isotype control	Rat	Alexa Fluor® 647	1:200	Biolegend	400526

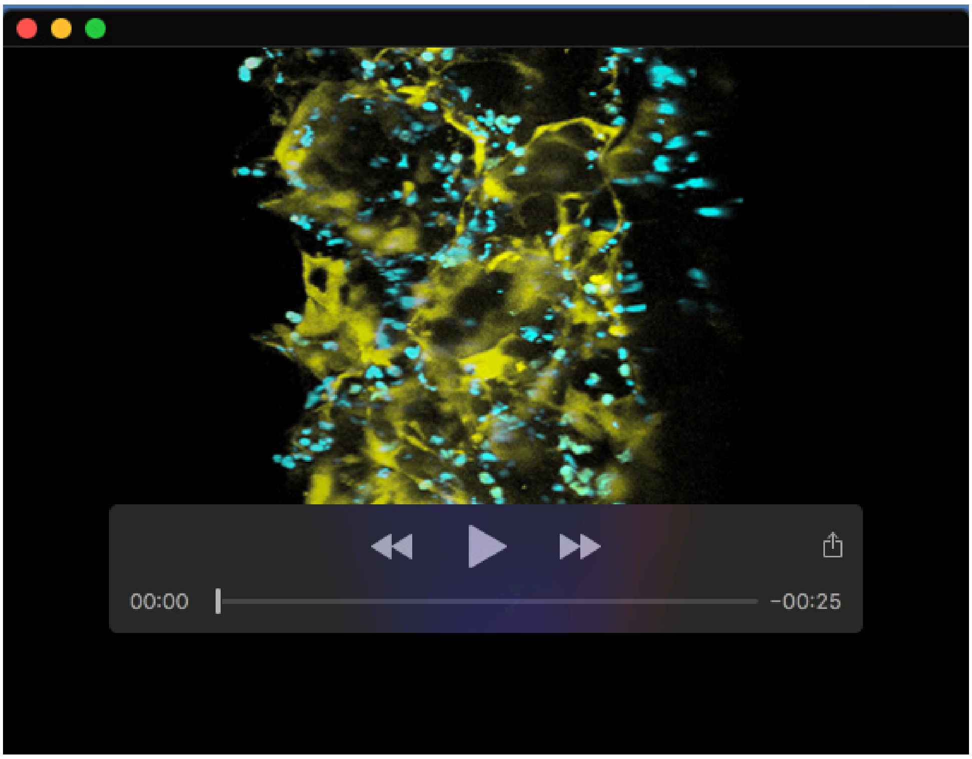

Movie 1. Video showing 3D projection of *R26R-EYFP* PCLS treated with TAT-Cre recombinase. A total of 126 Z-slices were acquired with 1 µm step size, reaching a depth of 125 µm. EYFP and DAPI signals are visualised as peak brightest point, rotating on the Y-axis. Images were captured on a confocal microscope using a HC PL APO 40×/1.30 oil objective lens (working distance = 0.24 mm). EYFP is shown in yellow and cell nuclei were counterstained with DAPI (cyan).
